# Individual and combined effects of *GSTM1* and *GSTT1* polymorphisms on colorectal cancer risk: an updated meta-analysis

**DOI:** 10.1042/BSR20201927

**Published:** 2020-08-25

**Authors:** Liang Song, Chen Yang, Xiao-Feng He

**Affiliations:** 1 Endoscopy Room, Heping Hospital Affiliated to Changzhi Medical College, Shanxi, Changzhi, 046000, People’s Republic of China; 2 Teaching Reform Class of 2016, First Clinical College, Changzhi Medical College, Shanxi, Changzhi, 046000, People’s Republic of China; 3 Department of Science and Education, Heping Hospital Affiliated to Changzhi Medical College, Shanxi, Changzhi, 046000, People’s Republic of China

**Keywords:** Colorectal cancer, GSTM1, GSTT1, meta analysis, Polymorphism

## Abstract

**Background.** The presence or absence of glutathione S-transferase M1 gene (*GSTM1*) and glutathione S-transferase T1 gene (*GSTT1*) polymorphisms, and their combined effects have been suggested as a risk factor for colorectal cancer (CRC). However, the results are inconsistent.

**Objectives.** An updated meta-analysis was performed to solve the controversy.

**Methods.** Meta-analyses of Observational Studies in Epidemiology (MOOSE) guidelines were used.

**Results.** Overall, the *GSTM1* null genotype was associated with an increased CRC risk in Caucasians (odds ratio (OR) = 1.14, 95% confidence interval (CI): 1.05–1.23), Asians (OR = 1.19, 95% CI: 1.08–1.32), high-quality studies (OR = 1.12, 95% CI: 1.06–1.18). Moreover, the *GSTM1* null genotype was also associated with an increased colon cancer risk (OR = 1.32, 95% CI: 1.16–1.51). The *GSTT1* null genotype was also associated with an increased CRC risk in Asians (OR = 1.08, 95% CI: 1.02–1.15) and Caucasians (OR = 1.24, 95% CI: 1.09–1.41). Moreover, The *GSTT1* null genotype was associated with an increased rectal cancer risk (OR = 1.13, 95% CI: 1.01–1.27, *I*^2^ = 8.3%) in subgroup analysis by tumor location. Last, the *GSTM1* null/*GSTT1* null genotype was associated with an increased CRC risk in Asians.

**Conclusion.** This meta-analysis indicates that the *GSTM1* and *GSTT1* null genotypes are associated with increased CRC risk in Asians and Caucasians, and the *GSTM1* null/*GSTT1* null genotype was associated with increased CRC risk in Asians.

## Introduction

Colorectal cancer (CRC) is a common form of cancer, with more than 1.5 million new patients diagnosed every year worldwide [1]. It is a complex chronic disease whose development is affected by genetic and environmental factors [2,3]. CRC incidence rates differ between countries indicating that environmental factors may be associated with an increased cancer risk, although. A twin study indicated that the role of genetic factors is around 35% in CRC [4]. A previous genome-wide association study also indicated that single-nucleotide polymorphisms are important risk factors [5].

Glutathione S-transferases (*GSTs*) are a large family of enzymes that catalyze the conjugation of electrophiles to glutathione and the conversion of toxic compounds to hydrophilic metabolites [6,7]. *GSTM1* maps to chromosome 1p13.3 contains 10 exons, while *GSTT1* maps to chromosome 22q11.23 and contains six exons. *GSTM1* present/null and *GSTT1* present/null polymorphisms have been reported in human [8–11]. The null genotypes are the most common polymorphisms in *GSTM1* and *GSTT1*, and have been proven to be associated with the loss of enzyme activity [12,13].

To date, many studies have evaluated the association between *GSTM1* present/null and *GSTT1* present/null polymorphisms, and their combined effects with CRC risk [14–107,108–114]. Additionally, 13 meta-analyses [115–125,126,127] have been conducted. However, a lot of studies have been published on these associations with CRC risk, therefore, an updated meta-analysis was performed to explore the association between *GSTM1* present/null, *GSTT1* present/null, and their combined effects on CRC risk in all populations.

## Materials and methods

### Search strategy

Meta-analyses of Observational Studies in Epidemiology (MOOSE) guidelines were used [128]. PubMed, Chinese Biomedical Medical databases (CBM), China National Knowledge Infrastructure (CNKI), and WanFang databases (up to March 15, 2020) were searched to identify eligible studies that analyzed the *GSTM1* present/null, *GSTT1* present/null, and their combined effects with CRC risk. The following keywords were used: (*GSTT1* OR glutathione S-transferase T1 OR *GSTM1* OR glutathione S-transferase M1) AND (polymorphism OR variant OR mutation) AND (colorectal OR rectal OR rectum OR colon). The search strategy was designed to be sensitive and broad. We first carefully reviewed the title and abstract of the search results, and then downloaded full articles to identify possible articles. These were evaluated in detail to identify relevant articles. The reference lists of identified articles and reviews was also examined as appropriate. The corresponding author may be contacted by e-mail if only the abstract was available online or the data was incomplete.

### Inclusion and exclusion criteria

Inclusion criteria were as follows: (1) articles on the *GSTM1* present/null, *GSTT1* present/null, and their combined effects with CRC risk; (2) sufficient genotype data to calculate ORs and 95% CIs; and (3) case–control studies. Exclusion criteria were as follows: (1) no raw data; (2) no control; (3) review articles, case reports, editorials, or animal research; (4) duplicate and insufficient data.

### Data extraction and quality score assessment

Two investigators independently extracted data using Excel. Any disagreement was solved by iteration, discussion, and consensus. The following data were extracted from eligible studies: (1) first author’s name, (2) publication year, (3) country, (4) source of controls (hospital-based and population-based case–control studies), (5) sample size, (6) genotyping method, and (6) genotype distribution of the *GSTM1, GSTT1*, and their combined effects in cases and controls. Different ethnicities included “Caucasians”, “Asians”, “Indians”, and “Africans”. If ethnicity was not stated or if the sample size could not be separated, the term “Mixed populations” was used. Two investigators independently assessed the quality of each individual study. The quality assessment criteria (Table 1) were obtained from two previous meta-analyses [129,130]. The highest value is obtained from the quality assessment was nine; studies of quality scoring ≥ 6 were considered as high quality.

**Table 1:** Scale for quality assessment

Criteria	Score
Representativeness of cases	
Selected from cancer registry or multiple cancer center sites	2
Selected from oncology department or cancer institute	1
Selected without clearly defined sampling frame or with extensive inclusion/exclusion criteria	0
Source of controls	
Population or community based	2
Both population-based and hospital-based/healthy volunteers/blood donors	1.5
Hospital-based controls without colorectal cancer	1
Cancer-free controls without total description	0.5
Not described	0
Ascertainment of colorectal cancer	
Histological or pathological confirmation	2
Diagnosis of colorectal cancer by patient medical record	1
Not described	0
Sample size	
>1000	2
200–1000	1
<200	0
Quality control of genotyping methods	
Clearly described a different genotyping assay to confirm the data	1
Not described	0

### Statistical analysis

We used crude odds ratios (ORs) and 95% confidence intervals (CIs) to estimate the association on the above issues. The genetic model of the individual *GSTM1* and *GSTT1* polymorphisms was null vs. present. Their combined effects used the following five genetic models: − − vs. + +, − − vs. + −, − − vs.− +, − − vs. (+ −) + (− +), and − − vs. (+ −) + (− +) + (+ +). − − referred to the *GSTM1* null/*GSTT1* null genotype, + − referred to the *GSTM1* present/*GSTT1* null genotype, − + referred to the *GSTM1* null/*GSTT1* present genotype, and + + referred to the *GSTM1* present/*GSTT1* present genotype. Heterogeneity among studies was tested using the *I*^2^ value [131]. A fixed-effects model (Mantel–Haenszel method) was used when *I*^2^ ≤ 50% [132]; otherwise, a random-effects model (DerSimonian and Laird method) was considered [133] if *I*^2^ > 50%. However, these studies cannot be pooled into together when *I*^2^ value > 75%. Subgroup analyses were performed by ethnicity, source of controls, tumor location, smoking history, gender, quality score, and tumor site. Then, a sensitivity analysis was carried out to assess the stability, a single study was excluded one at a time. Publication bias was tested by using Begg’s funnel and Egger’s test (significant publication bias was considered if *P* < 0.05). A nonparametric “trim and fill” method was applied to accredit missing studies if publication bias was detected. Finally, a meta-regression analysis was applied to assess the heterogeneity source. All results were calculated using Stata version 9.0 (Stata Corporation, College Station, TX, U.S.A.).

## Results

### Study characteristics

**Figure 1:**
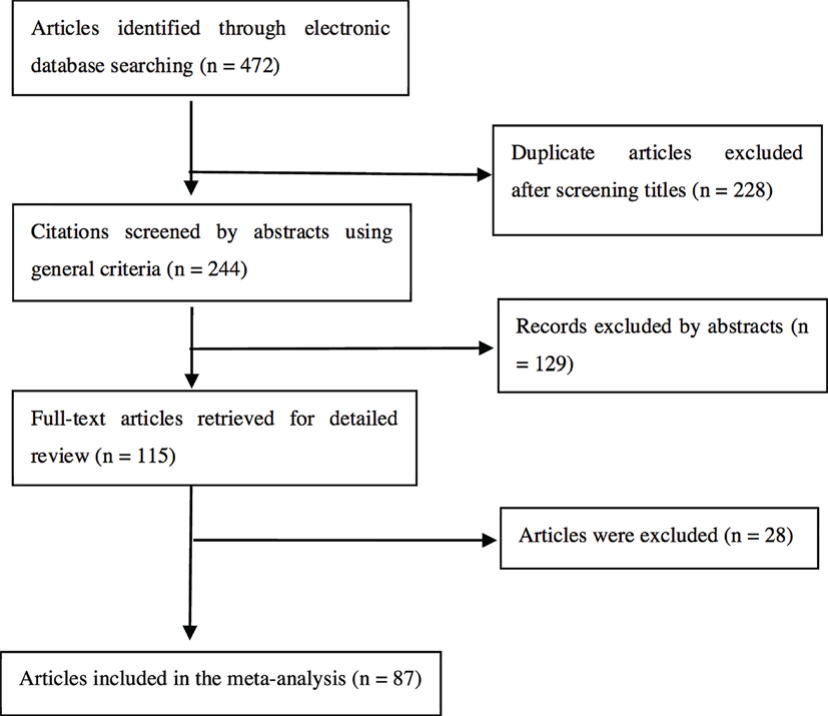
Flow diagram for identifying and including studies in the current meta-analysis

A flowchart of study selection is shown in Figure 1. Overall, 472 articles were identified by electronic database searching. Of these, 115 full-text articles were selected after carefully screening titles and abstracts. Fourteen articles were excluded because they were not case-control studies, while the data of fourteen articles [18,25,37,43,61,65,79,84,86,92,94,95,100,110] overlapped with those of another nine articles [26,41,47,48,93,105,107,108,114]. Hence, a total of 87 articles were included in the present meta-analysis.

The main study characteristics are listed in Tables 2 and 3. Eighty-five publications involving eighty-six case–control studies [14–17,19–24,26–36,38–42,44–60,62–64,66–78,80–83,85,87,88,90,91,93,96–99,101,103–107,108,109,111–114] were included on the *GSTM1* present/null polymorphism (24,931 cases and 36,537 controls; 44 studies on Caucasians, 31 on Asians, one on Africans, one on Indians, and nine on mixed populations) with CRC risk. Sixty-three articles of sixy-four case–control studies [15–17,19,21–24,26,27,30,31,33,34,36,38–42,45,47,48–52,54–58,62–64,67–70,73,74,76–78,80–82,87–90,93,96–99,102,105,109,111–114] were eligible concerning the *GSTT1* present/null polymorphism (19,725 cases and 28,725 controls; 34 studies on Caucasians, 23 on Asians, one on Indians, one on Africans, and five on mixed populations) with CRC risk. Thirty-two publications of thirty-three case–control studies [15,19,22–24,27,31,33,38,39,41,42,45,49,52,55–57,63,67,68,70,76–78,90,96,97,99,105,109,112] were included regarding their combined effects (8270 cases and 14,381 controls; 11 studies on Caucasians, 17 on Asians, one on Indians, one on Africans, and three on mixed populations) with CRC risk. Fifty-five studies had a quality score ≥ 6 and the remaining 31 had a quality score < 6 regarding the *GSTM1* present/null polymorphism; 48 high-quality studies were examined and the remaining 16 were low-quality concerning the *GSTT1* present/null polymorphism; a total of 25 high-quality and eight low-quality studies were included on their combined effects with CRC risk.

**Table 2:** The data between the *GSTM1* and *GSTT1* polymorphisms and colorectal cancer risk

First author/Year	Country	Ethnicity	SC	Sample size (case/ control)	Genotyping methods	*GSTM1* genotype distribution				*GSTT1* genotype distribution				Quality scores
						Case		Control		Case		Control		
						Present	Null	Present	Null	Present	Null	Present	Null	
Stojkovic [111] 2019	Serbia	Caucasian	HB	509/399	Multiplex PCR	249	260	204	195	145	364	91	308	6
Rodrigues-Fleming [112] 2018	Brazil	Mixed	HB	232/738	Multiplex PCR and PCR-RFLP	100	132	385	353	192	40	573	165	6.5
Waś [113] 2018	Poland	Caucasian	HB	279/233	PCR	151	128	133	100	220	59	189	44	6
Klusek [114] 2018	Poland	Caucasian	HB	197/104	TaqMan	105	92	57	47	166	31	83	21	6
Gorukmez [49] 2016	Turkey	Caucasian	HB	92/116	Multiplex PCR	65	27	67	49	58	34	91	25	4
Khabaz [32] 2016	Saudi Arabia	Caucasian	HB	83/35	PCR	14	69	12	23	NA	NA	NA	NA	3
Zeng [99] 2016	China	Asian	HB	108/215	PCR	38	70	110	105	48	60	117	98	6
Djansugurova [34] 2015	Kazakhstan	Mixed	HB	249/245	Site-specific PCR	124	125	158	87	171	78	164	81	4.5
Cong [33] 2014	China	Asian	PB	264/317	Multiplex PCR	122	142	182	135	125	139	190	127	6
Procopciuc [85] 2014	Romania	Caucasian	HB	150/162	PCR-RFLP	60	90	97	65	NA	NA	NA	NA	6
Vogtmann [31] 2014	China	Asian	PB	340/673	Real-time PCR	134	201	259	379	164	173	350	318	8
Kassab [76] 2014	Tunisia	Caucasian	HB	147/128	Multiplex PCR	43	104	41	87	90	57	65	63	6
Saeed [14] 2013	Saudi Arabia	Caucasian	HB	100/79	PCR	98	2	79	0	NA	NA	NA	NA	5
Chirila [15] 2013	Romania	Caucasian	HB	19/19	Multiple PCR	14	5	15	4	15	4	16	3	3
Hezova [16] 2012	Czech	Caucasian	HB	197/218	Duplex PCR	97	100	117	101	157	40	179	39	6.5
Rudolph [17] 2012	German	Caucasian	PB	1796/1806	Multiplex PCR	822	932	844	923	1433	313	1459	308	6
Huang [96] 2012	China	Asian	HB	130/100	PCR	71	59	58	42	63	67	52	48	6
Darazy [20] 2011	Lebanese	Caucasian	HB	67/70	PCR	32	25	58	12	NA	NA	NA	NA	3.5
Wang [23] 2011	India	Indian	HB	302/291	Multiplex PCR	202	100	215	76	245	57	247	44	6
Koh [19] 2011	China	Asian	PB	480/1167	TaqMan	246	234	641	526	294	186	691	476	8
Cleary [21] 2010	Canada	Caucasian	PB	1174/1293	Multiplex PCR	550	616	608	684	953	213	1,067	223	9
Yang [24] 2010	China	Asian	PB	322/1251	Real-time PCR	133	189	521	730	158	164	639	612	8
Nisa [78] 2010	Japan	Asian	PB	685/778	Multiplex PCR	328	357	356	422	347	338	435	343	8
Zhang SS [50] 2010	China	Asian	PB	197/399	Multiplex PCR	83	114	184	215	150	47	310	89	6
Hlavata [22] 2010	Czech	Caucasian	HB	495/495	PCR-RFLP	228	267	254	241	392	103	395	100	6
Csejtei [26] 2009	Hungary	Caucasian	HB	102/97	PCR	42	60	51	46	68	34	77	20	4
Piao [77] 2009	Korea	Asian	PB	1829/1699	Real-time PCR	825	1,004	776	923	879	950	841	858	9
Matakova [27] 2009	Slovak	Caucasian	PB	183/402	PCR	83	100	202	220	142	41	329	93	6
Zupa [28] 2009	Italy	Caucasian	HB	92/121	PCR	31	61	53	68	NA	NA	NA	NA	5
Curtin [29] 2009	U.S.A.	Caucasian	PB	750/1201	PCR	310	323	465	545	NA	NA	NA	NA	8
Epplein [30] 2009	U.S.A.	Mixed	PB	173/313	TaqMan	82	91	166	147	127	46	201	112	7
Lin LM [93] 2008	China	Asian	HB	120/204	Multiplex PCR	51	69	114	90	56	64	119	85	6
Yang ZF [46] 2008	China	Asian	HB	84/112	PCR	24	60	61	51	NA	NA	NA	NA	5
Cotterchio [88] 2008	Canada	Caucasian	PB	836/1249	Multiplex PCR	395	441	588	661	679	157	1,029	219	8
Kury [87] 2008	France	Caucasian	PB	1023/1121	TaqMan	479	544	553	568	840	183	916	205	8
Skjelbred [36] 2007	Norway	Caucasian	PB	108/299	Multiplex PCR	53	55	148	151	93	15	262	37	6
Yoshida [35] 2007	Japan	Asian	PB	66/121	PCR	30	36	59	62	NA	NA	NA	NA	3
Xia [59] 2007	China	Asian	HB	112/140	PCR	45	67	77	63	NA	NA	NA	NA	6
Huang [105] 2007	China	Asian	HB	57/68	PCR	17	40	33	35	33	24	44	24	5
Martínez [38] 2006	Spain	Caucasian	PB	144/329	Multiplex PCR	55	87	180	149	68	74	253	76	6
Probst-Hensch [39] 2006	China	Asian	PB	300/1169	TaqMan	168	132	643	525	200	100	693	475	9
Little [40] 2006	U.K.	Caucasian	PB	241/383	PCR	110	131	162	221	192	49	318	65	7
Fan [41] 2006	China	Asian	PB	140/343	PCR	58	80	151	188	113	25	270	69	6
Huang [42] 2006	U.S.A.	Caucasian	PB	315/547	Multiplex PCR	135	180	258	289	241	74	385	162	6
Huang [42] 2006	U.S.A.	African	PB	239/327	Multiplex PCR	162	77	245	82	187	56	218	109	6
Fu [98] 2006	China	Asian	PB	315/439	PCR	86	229	117	321	141	174	187	251	7
Luo [91] 2006	China	Asian	HB	56/143	PCR	36	20	95	48	NA	NA	NA	NA	3
Rajagopal [89] 2005	U.K.	Caucasian	HB	361/881	PCR	NA	NA	NA	NA	265	96	723	158	7
Landi [44] 2005	Spain	Caucasian	HB	176/162	PCR	77	99	66	96	NA	NA	NA	NA	4
Ateş [45] 2005	Turkey	Caucasian	HB	181/204	Real-Time PCR	83	98	116	88	118	63	151	53	6
Yeh [47] 2005	China	Asian	HB	727/736	Multiplex PCR	325	402	326	410	331	396	376	360	7
van der Logt [51] 2004	U.S.A.	Caucasian	PB	371/415	PCR	186	184	212	203	299	72	346	69	4
Kiss [48] 2004	Hungary	Caucasian	HB	500/500	PCR	209	291	258	242	369	131	392	108	6
Chen [109] 2004	China	Asian	HB	125/339	PCR	56	69	151	188	102	23	270	69	7
Smits [53] 2003	Multiple	Caucasian	PB	724/1743	PCR	381	343	821	922	NA	NA	NA	NA	7.5
van der Hel [54] 2003	U.S.A.	Caucasian	PB	212/765	PCR	124	88	396	369	154	58	541	224	6
Slattery [107] 2003	U.S.A.	Mixed	PB	801/1013	PCR	397	404	467	546	NA	NA	NA	NA	6
Nascimento [55] 2003	Brazil	Mixed	HB	102/300	Multiplex PCR	52	50	166	134	85	17	248	52	6
Huang [90] 2003	China	Asian	HB	82/82	Multiplex PCR	36	46	54	28	41	41	42	40	5
Yang [101] 2003	China	Asian	HB	58/65	PCR-RFLP	18	40	36	29	NA	NA	NA	NA	3
Zhang [102] 2003	China	Asian	HB	81/112	Multiplex PCR	NA	NA	NA	NA	27	54	54	58	5
Zhu [57] 2002	China	Asian	HB	104/101	Multiplex PCR	56	48	44	57	55	49	40	61	6
Ye [58] 2002	U.K.	Caucasian	HB	41/82	Specific PCR	21	20	49	33	39	2	73	9	5
Tiemersma [60] 2002	U.S.A.	Mixed	PB	102/537	PCR	44	58	252	285	NA	NA	NA	NA	6
Seow [56] 2002	China	Asian	PB	213/1194	TaqMan	105	108	653	537	133	80	710	480	9
Sachse [81] 2002	U.K.	Caucasian	PB	490/593	PCR	206	284	291	302	306	184	378	215	6
Laso [82] 2002	Spain	Caucasian	HB	247/296	Multiplex PCR	114	133	138	158	116	131	263	33	6
Sgambato [83] 2002	Italy	Caucasian	HB	44/100	Duplex PCR	12	32	47	53	NA	NA	NA	NA	3
Slattery [108] 2002	U.S.A.	Mixed	PB	1577/1904	PCR	761	816	892	1012	NA	NA	NA	NA	9
Butler [62] 2001	Australia	Caucasian	PB	219/200	PCR	97	106	92	108	123	67	160	40	4
Saadat [63] 2001	Iran	Caucasian	HB	46/131	PCR	21	25	78	53	28	18	90	41	5
Loktionov [64] 2001	U.K.	Caucasian	HB	206/355	PCR	73	133	147	208	166	40	301	54	6
Zhang [103] 2001	China	Asian	HB	52/52	Multiplex PCR	30	22	27	25	NA	NA	NA	NA	5
Zhou [97] 2000	China	Asian	HB	55/62	PCR	21	34	29	33	24	31	31	31	5
Gawrońska-Szklarz [66] 1999	Poland	Caucasian	HB	70/145	PCR	24	46	73	72	NA	NA	NA	NA	6
Yoshioka [67] 1999	Japan	Asian	HB	106/100	PCR	50	56	58	42	55	51	59	41	6
Abdel-Rahman [68] 1999	Egypt	Caucasian	HB	66/55	PCR	26	37	15	30	37	22	30	21	4
Zhang [80] 1999	Sweden	Caucasian	HB	94/109	Multiplex PCR	50	44	54	55	44	50	87	22	6
Welfare [69] 1999	U.K.	Caucasian	PB	196/178	PCR	94	102	88	90	157	39	148	30	6
Gao [104] 1998	China	Asian	HB	19/70	PCR	12	7	45	25	NA	NA	NA	NA	5
Lee [71] 1998	Singapore	Asian	HB	300/183	NA	172	128	94	89	NA	NA	NA	NA	4
Gertig [70] 1998	U.S.A.	Mixed	PB	212/221	PCR	97	114	104	117	173	36	169	51	7
Guo [72] 1996	China	Asian	HB	19/23	PCR	12	7	17	6	NA	NA	NA	NA	5
Katoh [73] 1996	Japan	Asian	HB	103/126	Multiplex PCR	47	56	71	55	53	50	70	56	4
Deakin [52] 1996	U.K.	Caucasian	HB	252/577	PCR	117	135	261	316	189	63	415	94	4
Chenevix-Trench [74] 1995	Australia	Caucasian	HB	132/200	NA	68	64	99	101	79	15	125	23	2
Zhong [75] 1993	U.K.	Caucasian	PB	196/225	PCR	86	110	131	94	NA	NA	NA	NA	4
Strange [106] 1991	U.K.	Mixed	HB	19/502	HSE	5	14	249	253	NA	NA	NA	NA	5

Abbreviations: HB, hospital-based study; HSE, horizontal starch gel electrophoresis; PB, population-based study; SC, source of control.

**Table 3:** The data between combined effects of *GSTM1* and *GSTT1* polymorphisms and colorectal cancer risk

First author/Year	Country	Ethnicity	SC	Sample size	+ −		− +		− −		+ +		+ − or − +		+ +, + −, or − +		Quality scores
				Case / control	Cases	Controls	Cases	Controls	Cases	Controls	Cases	Controls	Cases	Controls	Cases	Controls	
Rodrigues-Fleming [112] 2018	Brazil	Mixed	HB	232/738	19	82	97	270	14	83	68	303	116	352	184	655	6.5
Gorukmez [49] 2016	Turkey	Caucasian	HB	92/116	31	11	24	35	3	14	34	56	55	46	89	102	4
Zeng [99] 2016	China	Asian	HB	108/215	25	64	35	71	35	34	13	46	60	135	73	181	6
Kassab [76] 2014	Tunisia	Caucasian	HB	147/128	NA	NA	NA	NA	45	26	NA	NA	NA	NA	102	102	6
Cong [33] 2014	China	Asian	PB	264/317	22	54	23	44	119	83	100	136	45	98	145	234	6
Vogtmann [31] 2014	China	Asian	PB	332/633	NA	NA	NA	NA	106	169	67	128	159	336	226	464	8
Chirila [15] 2013	Romania	Caucasian	HB	19/19	NA	NA	NA	NA	2	3	3	15	14	1	17	16	3
Huang [96] 2012	China	Asian	HB	130/100	NA	NA	NA	NA	15	12	46	42	NA	NA	115	88	6
Wang [23] 2011	India	Indian	HB	302/291	42	37	85	69	15	7	160	178	127	106	287	284	6
Koh [19] 2011	China	Asian	PB	480/1167	NA	NA	NA	NA	163	421	108	263	209	483	317	746	8
Yang [24] 2010	China	Asian	PB	322/1247	NA	NA	NA	NA	96	326	65	234	161	687	226	921	8
Nisa [78] 2010	Japan	Asian	PB	685/778	NA	NA	NA	NA	183	189	NA	NA	NA	NA	502	589	8
Hlavata [22] 2010	Czech	Caucasian	HB	495/495	NA	NA	NA	NA	61	46	186	200	248	249	434	449	6
Piao [77] 2009	Korea	Asian	PB	1829/1699	428	391	477	456	533	467	391	385	905	847	1296	1232	9
Matakova [27] 2009	Slovak	Caucasian	PB	183/422	20	35	83	162	19	58	61	167	103	197	164	364	6
Huang [105] 2007	China	Asian	HB	57/68	3	13	19	24	19	24	14	20	22	37	36	57	5
Martínez [38] 2006	Spain	Caucasian	PB	142/329	NA	NA	NA	NA	40	24	21	128	81	177	102	305	6
Probst-Hensch [39] 2006	China	Asian	PB	300/1168	NA	NA	NA	NA	45	222	NA	NA	NA	NA	255	946	9
Fan [41] 2006	China	Asian	PB	138/339	5	33	60	152	20	36	53	118	65	185	118	303	6
Huang [42] 2006	U.S.A.	Caucasian	PB	315/547	36	79	142	206	38	83	99	179	178	285	277	464	6
Huang [42] 2006	U.S.A.	African	PB	239/327	37	82	58	55	19	27	125	163	95	137	220	300	6
Ateş [45] 2005	Turkey	Caucasian	HB	180/204	36	34	71	69	27	19	46	82	107	103	153	185	6
Chen [109] 2004	China	Asian	HB	125/339	5	32	51	152	18	35	51	119	56	184	107	303	7
Nascimento [55] 2003	Brazil	Mixed	HB	102/300	NA	NA	NA	NA	9	24	44	138	49	138	93	276	6
Huang [90] 2003	China	Asian	HB	82/82	15	26	20	14	26	14	21	28	35	40	56	68	5
Zhu [57] 2002	China	Asian	HB	104/101	35	37	31	36	28	11	10	17	66	73	76	90	6
Seow [56] 2002	China	Asian	PB	213/1190	NA	NA	NA	NA	39	224	NA	NA	NA	NA	174	966	9
Saadat [63] 2001	Iran	Caucasian	HB	46/131	9	27	16	39	9	14	12	51	25	66	37	117	5
Zhou [97] 2000	China	Asian	HB	55/62	14	14	17	16	17	17	7	15	31	30	38	45	5
Yoshioka [67] 1999	Japan	Asian	HB	106/100	20	22	25	23	31	19	30	36	45	45	75	81	6
Abdel-Rahman [68] 1999	Egypt	Caucasian	HB	56/49	10	4	18	17	12	17	16	11	28	21	44	32	4
Gertig [70] 1998	U.S.A.	Mixed	PB	208/220	NA	NA	NA	NA	24	23	83	75	101	122	184	197	7
Deakin [52] 1996	U.K.	Caucasian	HB	218/448	38	37	89	207	26	42	65	162	127	244	192	406	4

Abbreviations: HB hospital-based studies; NA not available; PB population-based studies; SC, source of controls.

### Meta-analysis results

#### *GSTM1* present/null polymorphisms

**Figure 2:**
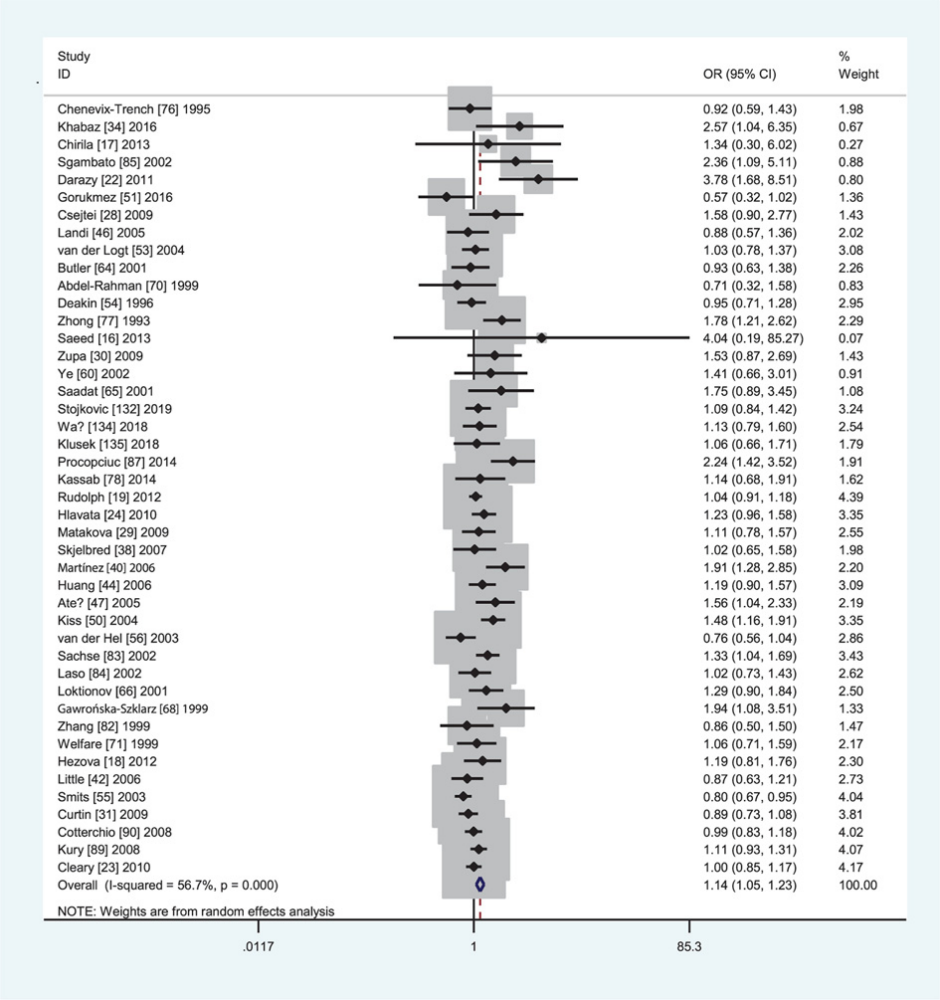
Forest plot of the association between *GSTM1* present/null polymorphism and CRC risk in Caucasians

**Figure 3:**
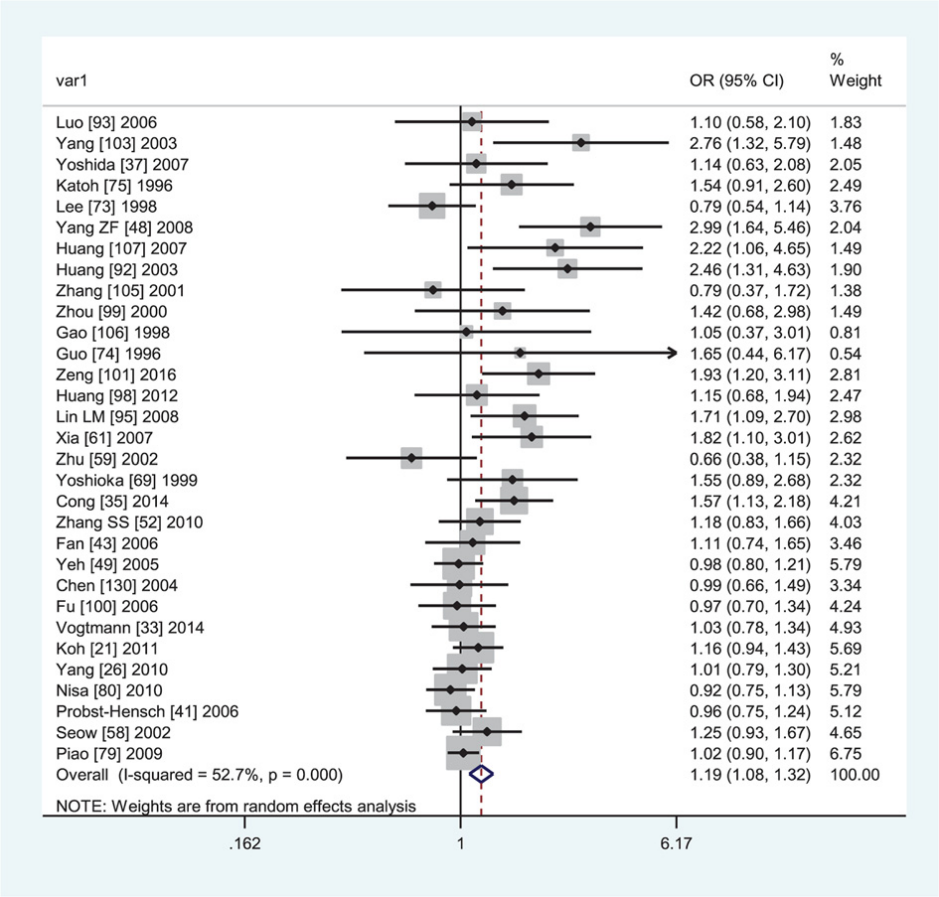
Forest plot of the association between *GSTM1* present/null polymorphism and CRC risk in Asians

Table 4 lists the summary ORs and 95% CIs on the *GSTM1* null genotype with CRC risk. The *GSTM1* null genotype was associated with an increased CRC risk (OR = 1.17, 95% CI: 1.10–1.23, *I*^2^ = 55.8%) in the overall population. In subgroup analyses by ethnicity, source of controls, and quality score, a significantly increased CRC risk was observed in Caucasians (OR = 1.14, 95% CI: 1.05–1.23, *I*^2^ = 56.7%, Figure 2) and Asians (OR = 1.19, 95% CI: 1.08–1.32, *I*^2^ = 52.7%, Figure 3), hospital-based studies (OR = 1.32, 95% CI: 1.20–1.46, *I*^2^ = 51.4%), high-quality studies (OR = 1.12, 95% CI: 1.06–1.18, *I*^2^ = 50.7%) and low quality studies (OR = 1.38, 95% CI: 1.17–1.62, *I*^2^ = 58.9%). Moreover, the *GSTM1* null genotype was also associated with an increased colon cancer risk (OR = 1.32, 95% CI: 1.16–1.51, *I*^2^ = 57.7%).

**Table 4:** Odds ratios and 95% confidence intervals for the association between *GSTM1* present/null polymorphism and colorectal cancer

Variable	No. of studies	No. of cases/controls	No. of *GSTM1* null cases/controls	Test of association				Test of heterogeneity	
				OR	95% CI	*Z*	*P*	Chi-square	*I*^2^ (%)
Overall	86	24,931/36,537	13,180/18,518	**1.17**	1.10–1.23*	5.24	<0.001	192.37	55.8
Ethnicity									
Caucasian	44	13,363/17,720	7073/9042	**1.14**	1.05–1.23*	3.22	0.001	99.37	56.7
Asian	31	7561/12,426	4126/6384	**1.19**	1.08–1.32*	3.39	0.001	63.44	52.7
Source of controls									
HB	51	7892/10,179	4168/4867	**1.32**	1.20–1.46*	5.59	<0.001	102.97	51.4
PB	35	17,039/26,358	9012/13,651	1.03	0.99–1.07	1.30	0.195	59.04	42.4
Quality score									
≥6	55	21,644/32,009	11,484/16,403	**1.11**	1.05–1.18*	3.84	<0.001	109.44	50.7
<6	31	3287/4528	1696/2115	**1.38**	**1.17–1.62**	3.78	<0.001	73.05	58.9
Location									
Colon cancer	23	5020/9672	2674/4728	**1.32**	1.16–1.51*	4.13	<0.001	51.95	57.7
Rectal cancer	15	3696/9355	1787/4544	0.99	0.91–1.07	0.27	0.79	21.72	35.5
Smoking									
Smokers	16	3444/4007	1778/2027	1.03	0.94–1.13	0.56	0.572	20.26	26.0
Non-smokers	15	2722/4177	1344/2083	1.05	0.87–1.25*	0.56	0.578	37.26	62.4
Gender									
Males	9	3410/4132	1832/2226	1.02	0.93–1.11	0.32	0.748	12.07	33.7
Females	9	2607/3905	1424/2198	0.99	0.85–1.17	0.08	0.932	15.14	47.2
Site									
Distal	10	1631/4017	880/2075	1.26	0.98–1.63*	1.81	0.071	30.55	70.5
Proximal	10	1246/4017	631/2075	1.03	0.78–1.36*	0.21	0.832	24.41	63.1

#### *GSTT1* present/null polymorphisms–

**Figure 4:**
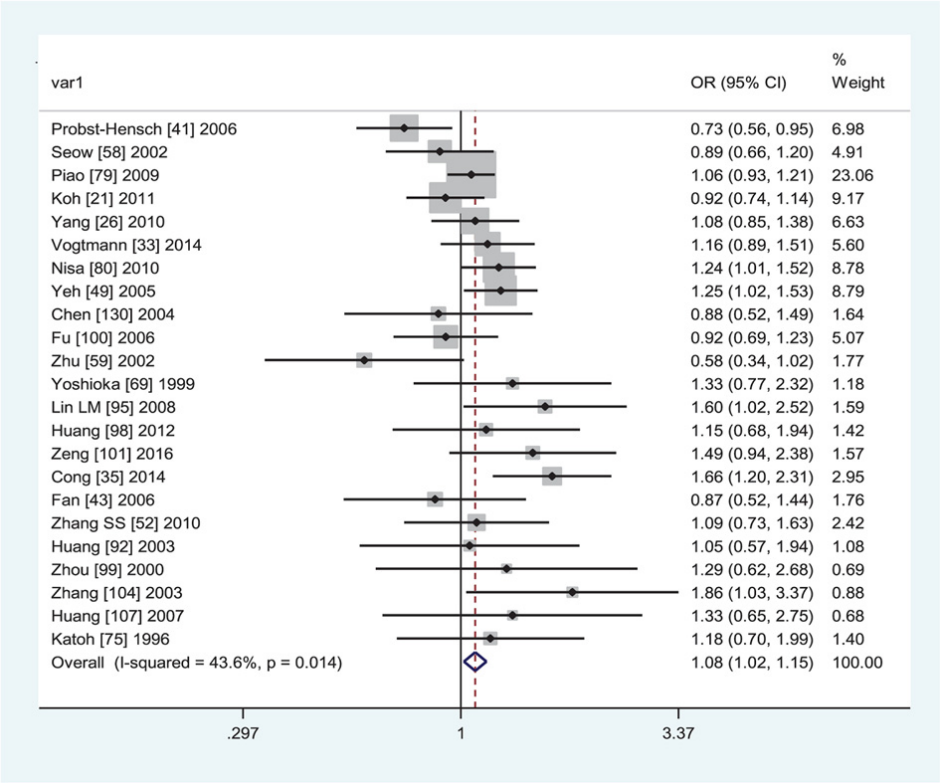
Forest plot of the association between *GSTT1* present/null polymorphism and CRC risk in Asians

Table 5 lists the summary ORs and 95% CIs on the *GSTT1* null genotype with CRC risk. The included studies could not be merged together because *I*^2^ > 75% was found between the *GSTT1* present/null polymorphism and CRC risk in the overall analysis and Caucasians. In subgroup analysis by ethnicity and quality score, a significantly increased CRC risk was observed in Asians (OR = 1.08, 95% CI: 1.02–1.15, *I*^2^ = 43.6%, Figure 4) and low-quality studies (OR = 1.33, 95% CI: 1.16–1.53, *I*^2^ = 17.3%). The *GSTT1* null genotype was also associated with an increased rectal cancer risk (OR = 1.13, 95% CI: 1.01–1.27, *I*^2^ = 8.3%) in subgroup analysis by tumor location.

**Table 5:** Odds ratios and 95% confidence intervals for the association between *GSTT1* present/null polymorphism and colorectal cancer risk

Variable	No. of studies	No. of cases/controls	No. of GSTT1 null cases/controls	Test of association				Test of heterogeneity	
				OR	95% CI	*Z*	*P*	Chi-square	*I*^2^ (%)
Overall	64	19,725/28,725	6512/8888	–	–	–	–	260.28	75.8
Ethnicity									
Caucasian	34	11,337/14,632	2896/3205	–	–	–	–	188.52	82.5
Asian	23	6878/11,659	3286/5069	**1.08**	**1.02–1.15**	2.49	0.013	39.03	43.6
Source of controls									
HB	36	6801/8894	2459/2552	–	–	–	–	154.05	77.3
PB	28	12,924/19,831	4053/6336	1.05	0.95–1.16*	0.96	0.337	90.02	70.0
Quality score									
≥6	48	17,832/26,262	5903/8253	–	–	–	–	234.52	80.0
<6	16	1893/2463	609/635	**1.33**	**1.16-1.53**	4.09	<0.001	18.14	17.3
Location									
Colon cancer	11	2324/6062	679/1889	1.11	0.94-1.32	1.22	0.224	16.48	39.3
Rectal cancer	10	2079/6661	695/2143	**1.13**	**1.01-1.27**	2.09	0.036	9.81	8.3
Smoking									
Smokers	12	2037/2405	537/641	1.04	0.83–1.30	0.36	0.721	21.46	48.7
Non-smokers	11	1730/2605	386/641	0.96	0.74–1.25	0.28	0.777	23.33	57.1
Gender									
Males	5	1930/2401	615/752	1.13	0.98–1.30	1.71	0.087	3.12	0.0
Females	5	1467/2436	493/930	1.10	0.95–1.28	1.24	0.217	3.51	0.0
Site									
Distal	7	723/1677	194/368	1.24	0.91–1.69	1.34	0.179	10.99	45.4
Proximal	7	340/1677	83/368	1.04	0.78–1.39	0.27	0.786	3.51	0.0

#### Combined effects of *GSTM1* and *GSTT1* present/null polymorphisms

Table 6 lists the summary ORs and 95% CIs on their combined effects with CRC risk. The *GSTM1* null/*GSTT1* null genotype was associated with an increased CRC risk in the overall analysis (− − vs. + +: OR = 1.42, 95% CI: 1.17–1.73, *I*^2^ = 68.6%; − − vs. + −: OR = 1.37, 95% CI: 1.00–1.88, *I*^2^ = 73.0%; − − vs. (+ −) + (− +): OR = 1.26, 95% CI: 1.05–1.51, *I*^2^ = 70.4%; − − vs. (+ −) + (− +) + (+ +): OR = 1.26, 95% CI: 1.09–1.46, *I*^2^ = 69.0%).

**Table 6:** Combined genotype analysis of the GSTM1 and GSTT1 polymorphisms on risk of colorectal cancer

Variables	No. of studies	No. of cases/controls	Test of association				Test of heterogeneity	
			OR	95% CI	*Z*	*P*	Chi-squared	*I*^2^ (%)
− − vs. + +								
Overall	29	3543/5647	**1.42**	1.17–1.73*	3.50	<0.001	89.24	68.6
Ethnicity								
Caucasian	10	780/1371	–	–	–	–	52.35	82.8
Asian	14	2202/3255	**1.41**	1.15–1.73*	3.29	0.001	28.51	54.4
Source of controls								
HB	18	1193/1954	**1.53**	**1.28–1.83**	4.66	<0.001	31.24	45.6
PB	11	2350/3337	–	–	–	–	51.81	80.7
Quality score								
≥ 6	21	3257/5144	**1.43**	**1.15–1.77**	3.19	0.001	75.95	73.7
< 6	8	286/503	1.38	0.85**–**2.24*	1.32	0.187	12.76	45.1
− − vs. − +								
Overall	20	2469/3221	1.15	0.92–1.44*	1.21	0.226	46.25	58.9
Ethnicity								
Caucasian	7	577/982	0.89	0.61–1.28*	0.64	0.522	11.35	47.1
Asian	10	1604/1728	**1.28**	**1.11–1.48**	3.42	0.001	17.16	47.6
Source of controls								
HB	14	878/1392	1.21	0.99–1.48	1.89	0.059	24.25	46.4
PB	6	1591/1829	–	–	–	–	20.28	75.3
Quality score								
≥ 6	13	2154/2727	1.20	0.91–1.60*	1.28	0.199	40.26	70.2
< 6	7	315/494	1.07	0.77–1.47	0.39	0.693	5.99	0.0
− − vs. + −								
Overall	20	1878/2218	**1.37**	1.00–1.88*	1.98	0.048	70.50	73.0
Ethnicity								
Caucasian	7	314/474	0.66	0.37–1.17*	1.42	0.154	18.89	68.2
Asian	10	1418/1426	–	–	–	–	36.61	75.4
Source of controls								
HB	14	582/790	1.32	0.83–2.09*	1.18	0.239	44.80	71.0
PB	6	1296/1428	–	–	–	–	24.47	79.6
Quality score								
≥ 6	13	1646/1944	**1.60**	1.15–2.22*	2.82	0.005	39.67	69.7
< 6	7	232/274	–	–	–	–	28.60	79.0
− − vs.(− +) + (+ −)								
Overall	28	4842/7564	**1.26**	1.05–1.51*	2.45	0.014	91.18	70.4
Ethnicity								
Caucasian	10	1203/1709	–	–	–	–	41.23	78.2
Asian	13	3070/4836	**1.50**	1.20–1.86*	3.60	<0.001	40.06	70.0
Source of controls								
HB	17	1563/2293	1.23	0.92–1.63*	1.40	0.162	39.44	59.4
PB	11	3279/5271	–	–	–	–	50.60	80.2
Quality score								
≥ 6	20	4391/6934	**1.33**	1.09–1.62*	2.85	0.004	71.8	73.6
< 6	8	451/630	0.91	0.53**–**1.54	0.36	0.715	18.80	62.8
− − vs.(− +) + (+ −) + (+ +)								
Overall	33	8270/14,381	**1.26**	1.09–1.46*	3.08	0.002	103.11	69.0
Ethnicity								
Caucasian	8	1893/2888	–	–	–	–	47.52	79.0
Asian	17	5328/9617	**1.30**	1.10–1.53*	3.14	0.002	47.75	66.5
Source of controls								
HB	19	2620/3998	**1.38**	**1.19-1.60**	4.17	<0.001	35.47	49.3
PB	14	5650/10,383	–	–	–	–	61.31	78.8
Quality score								
≥ 6	25	7647/13,393	**1.29**	1.10–1.51*	3.08	0.002	88.88	73.0
< 6	8	623/988	1.10	0.72–1.70*	0.45	0.656	14.27	0.047

+ −: *GSTM1* present/GSTT1 null; − +: *GSTM1* null/*GSTT1* present; − −: *GSTM1* null/*GSTT1* null; + +: *GSTM1* present/*GSTT1* present; HB Hospital-based studies; PB Population-based studies

**Figure 5:**
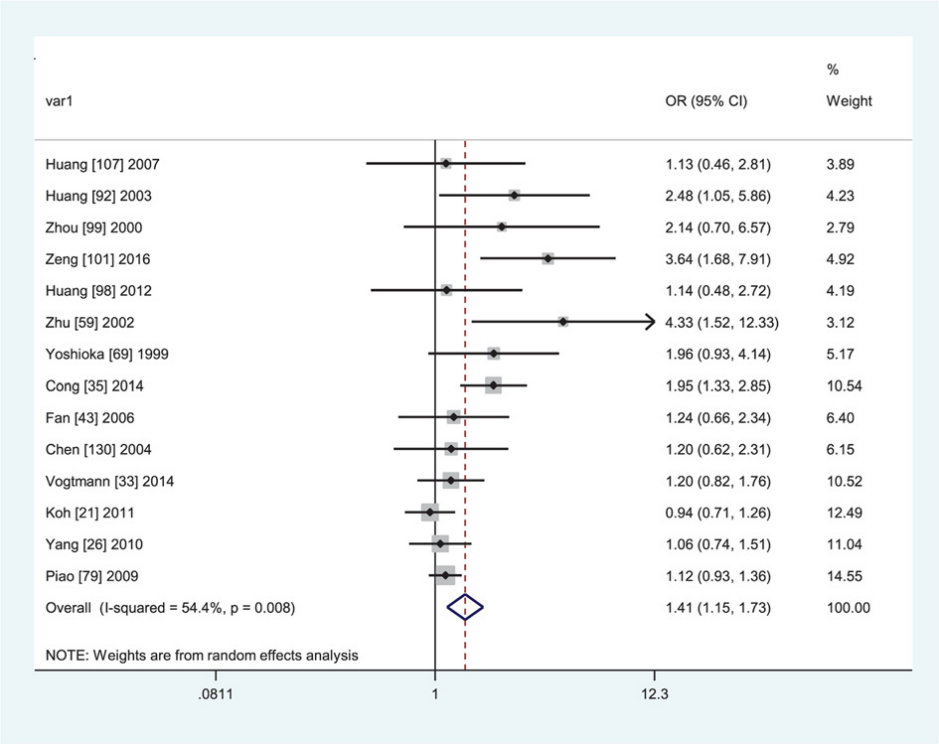
Forest plot of the association between the combined of *GTSM1* present/null and *GSTT1* present/null polymorphisms and CRC risk in Asians

In subgroup analyses by ethnicity, source of controls, and quality score, the *GSTM1* null/*GSTT1* null genotype was associated with an increased CRC risk in Asians (− − vs. + +: OR = 1.41, 95% CI: 1.15–1.73, *I*^2^ = 54.4%, Figure 5; − − vs. − +: OR = 1.28, 95% CI: 1.11–1.48, *I*^2^ = 47.6%; − − vs. (+ −) + (− +): OR = 1.50, 95% CI: 1.20–1.86, *I*^2^ = 70.0%; − − vs. (+ −) + (− +) + (+ +): OR = 1.38, 95% CI: 1.19–1.60, *I*^2^ = 49.3%), hospital-based studies (− − vs. + +: OR = 1.53, 95% CI: 1.28–1.83, *I*^2^ = 45.6%; − − vs. (+ −) + (− +) + (+ +): OR = 1.38, 95% CI: 1.19–1.60, *I*^2^ = 49.3%) and high-quality studies (− − vs. − +: OR = 1.43, 95% CI: 1.15–1.77, *I*^2^ = 73.7%; − − vs. + −: OR = 1.60, 95% CI: 1.16–2.22, *I*^2^ = 69.7%; − − vs. (+ −) + (− +): OR = 1.33, 95% CI: 1.09–1.62, *I*^2^ = 73.6%; − − vs. (+ −) + (− +) + (+ +): OR = 1.29, 95% CI: 1.10–1.51, *I*^2^ = 73.0%).

#### Heterogeneity and sensitivity analyses

Significant heterogeneity was detected in the meta-analysis, as shown in Tables 4–6. A meta-regression analysis revealed that sample size (*P*=0.002) was the source of heterogeneity for the *GSTM1* present/null polymorphism. Concerning the *GSTT1* present/null polymorphism and the combined effects of *GSTM1* and *GSTT1*, meta-regression analysis did not reveal a source of heterogeneity under any genetic model. Additionally, *I^2^* > 75% as shown in Tables 4–6.

**Figure 6:**
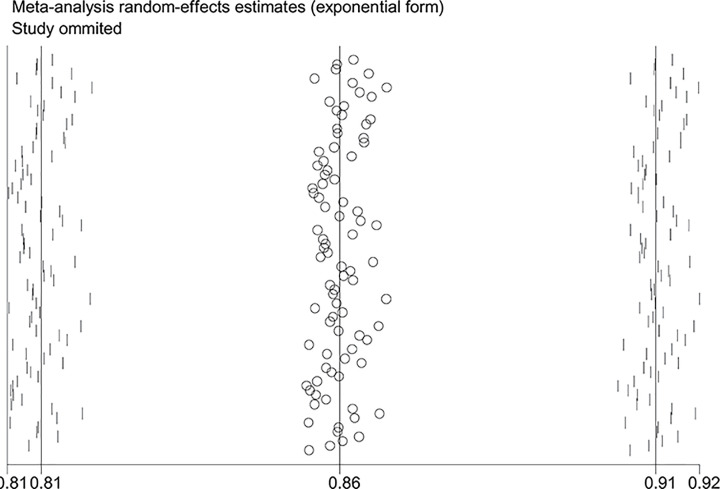
Sensitive analysis of the null genotype of *GSTM1* on CRC risk in overall population

**Figure 7:**
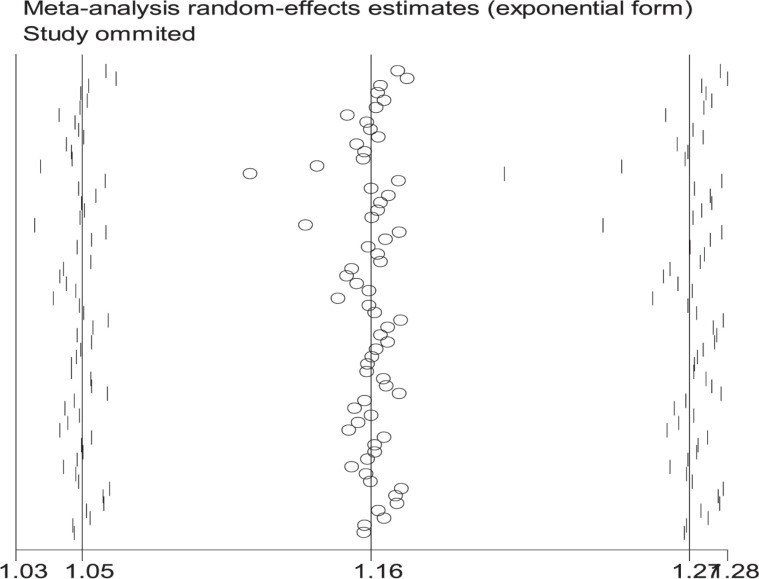
Sensitive analysis of the null genotype of *GSTT1* on CRC risk in overall population

**Figure 8:**
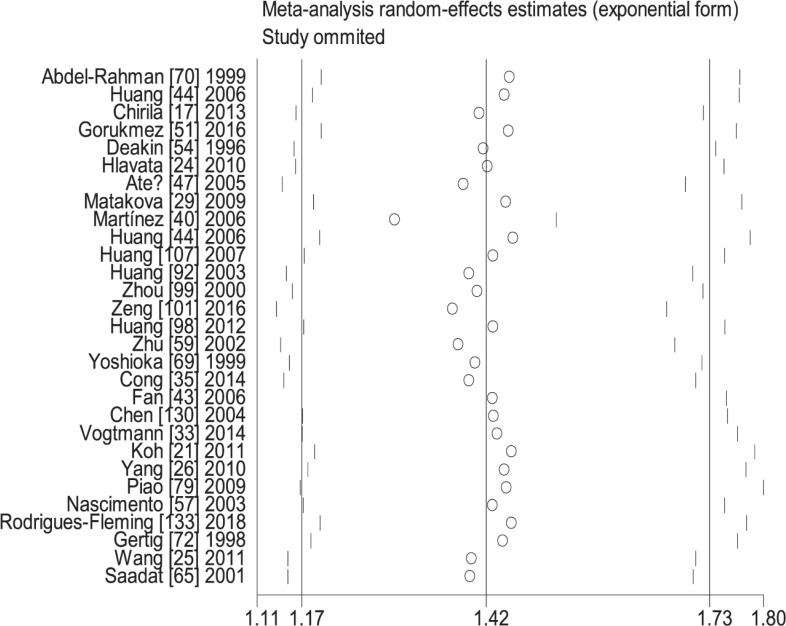
Sensitive analysis of the combined effects of *GSTM1* and *GSTT1* on CRC risk in overall population (− − vs. + +)

**Figure 9:**
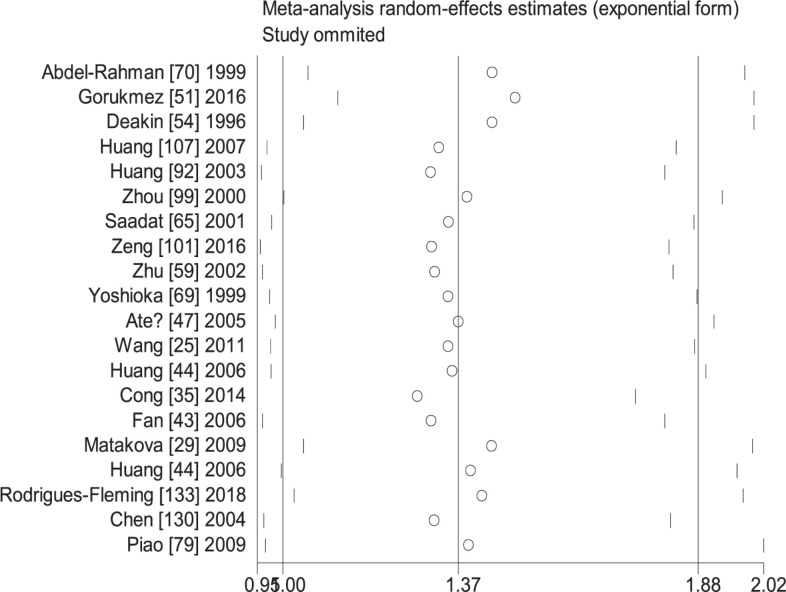
Sensitive analysis of the combined effects of *GSTM1* and *GSTT1* on CRC risk in overall population (− − vs. + −)

**Figure 10:**
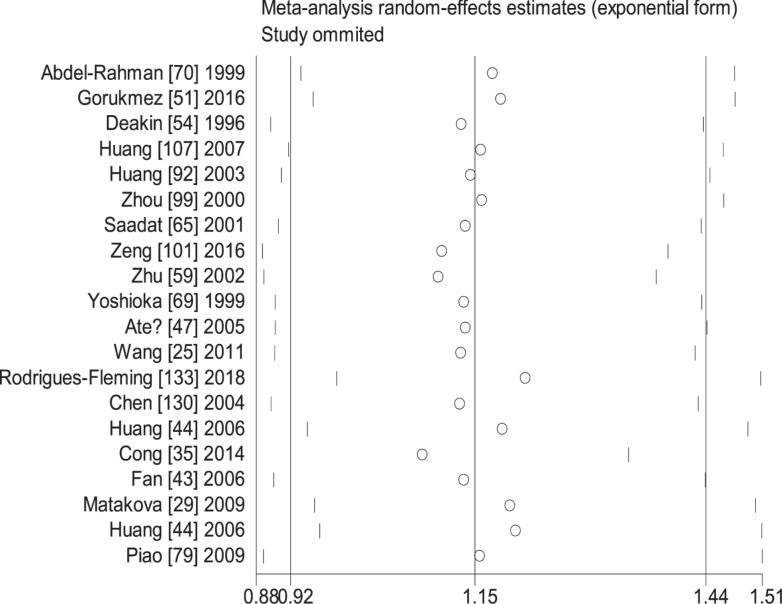
Sensitive analysis of the combined effects of *GSTM1* and *GSTT1* on CRC risk in overall population (− − vs. − +)

**Figure 11:**
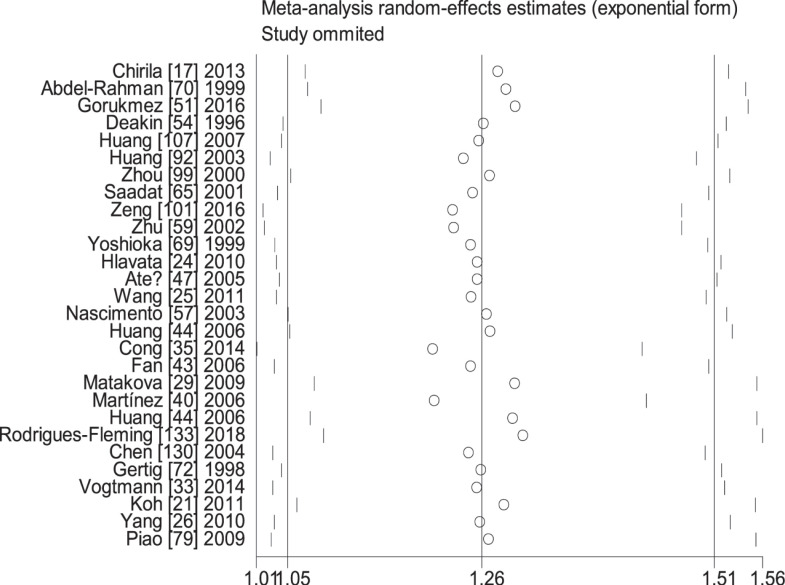
Sensitive analysis of the combined effects of *GSTM1* and *GSTT1* on CRC risk in overall population (− − vs. (+ −) + (− +))

**Figure 12:**
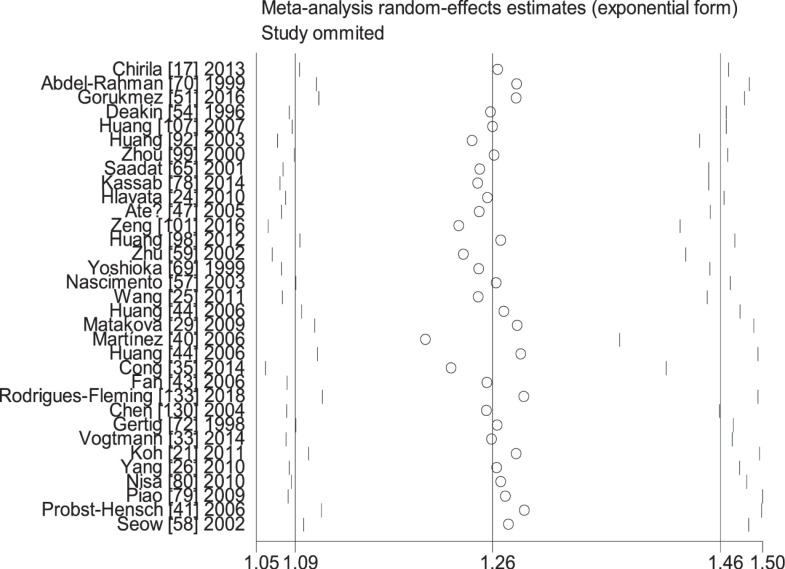
Sensitive analysis of the combined effects of *GSTM1* and *GSTT1* on CRC risk in overall population ((+ −) + (− +) + (+ +))

When the study of Laso et al. [82] was excluded, the values of heterogeneity dropped and the *GSTT1* null genotype was associated with an increased CRC risk in the following subgroups: Caucasians (OR = 1.24, 95% CI: 1.09–1.41, *I*^2^ = 70.8%) and hospital-based studies (OR = 1.19, 95% CI: 1.06–1.35, *I*^2^ = 54.5%). When the study of Martínez et al. [38] was excluded, the *I*^2^ value dropped and no significant association was found between the combined effects of *GSTM1* and *GSTT1* polymorphisms and CRC risk in Caucasians (− − vs. + +: OR = 1.22, 95% CI: 0.83–1.78, *I*^2^ = 55.6%; − − vs. (+ −) + (− +): OR = 0.81, 95% CI: 0.53–1.26, *I*^2^ = 68.1%; − − vs. (+ −) + (− +) + (+ +): OR = 0.99, 95% CI: 0.69–1.41, *I*^2^ = 57.0%) and population-based studies (− − vs. + +: OR = 1.11, 95% CI: 0.99–1.24, *I*^2^ = 28.9%; − − vs. (+ −) + (− +): OR = 1.16, 95% CI: 0.93–1.45, *I*^2^ = 73.9%; − − vs. (+ −) + (− +) + (+ +): OR = 1.08, 95% CI: 0.93–1.26, *I*^2^ = 63.5%). When the study of Gorukmez [49] was deleted, the *I*^2^ value dropped and no significant association was observed between the combined effects of *GSTM1* and *GSTT1* polymorphisms and CRC risk in low-quality studies (− − vs. + −: OR = 1.23, 95% CI: 0.58–2.59, *I*^2^ = 68.3%). When the study of Cong et al. [33] was excluded, the *I*^2^ value dropped and no significant association was observed between the combined effects of *GSTM1* present/null and *GSTT1* present/null polymorphisms and CRC risk in population-based studies (− − vs. + −: OR = 1.13, 95% CI: 0.78–1.65, *I*^2^ = 54.4%; − − vs. − +: OR = 0.88, 95% CI: 0.65–1.19, *I*^2^ = 55.3%). A single study was excluded each time to assess the stability of the results. Figures 6–12 suggest that the results are stable in the present meta-analysis.

#### Publication bias

**Figure 13:**
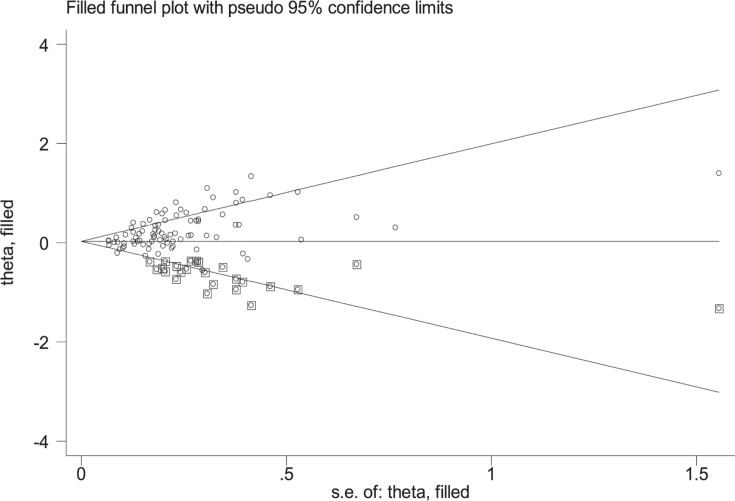
The Duval and Tweedie nonparametric “trim and fill” method’s funnel plot of the *GSTM1* present/null polymorphism

Begg's funnel plot and Egger's test were used to assess publication bias in the meta-analysis. The Begg's funnel plot shape and Egger’s test (*P*<0.001) revealed obvious publication bias between the *GSTM1* present/null polymorphism and CRC risk in the overall analysis. Figure 13 shows the Begg’s funnel plots by the trim and fill method; 24 missing studies should be added to this. Notably, log OR and 95% CI did not alter significantly when the trim and fill method was used. No significant publication bias was observed for the *GSTT1* present/null polymorphism (*P*=0.195). Concerning their combined effects, no publication bias was detected under any genetic model (*P*=0.093 for − − vs. + +; *P*=0.398 for − − vs. + −; *P*=0.764 for − − vs. − +; *P*=0.643 for − − vs. (+ −) + (− +); *P*=0.280 for − − vs. (+ −) + (− +) + (+ +)).

## Discussion

Strange et al. [106] in 1991 first reported an association between the *GSTM1* null genotype and colon adenocarcinoma risk. Chenevix-Trench et al. [21] first analyzed the association between the *GSTT1* null genotype and CRC risk in 1996. Deakin et al. [52] first examined their combined effects with CRC risk in 1996. Since then, many case-control studies have investigated the associations but the results are still inconsistent. Hence, an updated meta-analysis was performed to explore the *GSTM1* null genotype, *GSTT1* null genotype, and their combined effects with CRC risk.

Overall, this meta-analysis indicates that the *GSTM1* and *GSTT1* null genotypes are associated with increased CRC risk in Asians and Caucasians, and the *GSTM1* null/*GSTT1* null genotype was associated with increased CRC risk in Asians, but not in Africans and Indians. In addition, the *GSTM1* null genotype was associated with colon cancer risk but not rectal cancer, while conversely that the *GSTT1* null genotype was associated with rectal caner but not colon cancer.

Actually, it may not be uncommon that the same polymorphism played different roles in cancer risk among different ethnic population, because cancer is a complicated multi-genetic disease, and different genetic backgrounds may contribute to the discrepancy [134]. In addition, the differences might arise by chance because studies in Indians and Africans with small sample size may have insufficient statistical power to generate an authoritative risk estimate [135]. Therefore, a large population-based case-control study is required to confirm the GSTM1, GSTT1 and their combined effects with CRC risk in Indians and Africans. Nine [32,33,46,59,90,93,99,101,105] and seven [38,45,48,75,81,83,85] studies indicated that the *GSTM1* null genotype was associated with an increased CRC risk in Asians and Caucasians, respectively. Five [33,47,78,93,102] and eight [26,38,49,52,62,80,82,89] studies indicated that the *GSTT1* null genotype had a significantly increased CRC risk in Asians and Caucasians, respectively. Moreover, five studies [33,41,57,90,99] reported a significant association between their combined effects and CRC risk in Asians. The results of present study strongly supported these findings.

Subgroup analysis by source of control found a significant association in hospital-based studies, but not in population-based studies in the present meta-analysis. However, hospital-based controls are not likely to replace the general population because they may have more bias than population-based studies [136]. Therefore, the results of hospital-based controls should be carefully explained. Heterogeneity is a common problem in meta-analyses. The present study observed several high levels of heterogeneity (*I^2^* > 75%), and the results of meta-regression analysis indicated that sample size was the source of heterogeneity between the *GSTM1* null genotype and CRC risk. Small sample size studies may be important confounding bias in molecular epidemiological studies, because random error and bias were common in the studies with small sample sizes, and the results were unreliable [137]. Furthermore, small sample studies were easier to accept if there was a positive report as they tend to yield false-positive results because they may be not rigorous and are often of low-quality. In addition, several value of *I^2^* > 75% dropped when a single study was excluded, the results indicate that source of heterogeneity also may be from one or multiple small sample or low quality studies. Figure 13 indicates that the asymmetry of the funnel plot was caused by studies with low-quality small samples.

A total of 13 meta-analyses [115–125,126,127] were conducted between 2010 and 2019 reported on the associations between the *GSTM1* present/null and/or *GSTT1* present/null polymorphisms with CRC risk. Cai et al. [115] examined 17 studies that included 5907 CRC cases and 9726 controls to explore the association between the *GSTM1* null genotype and CRC risk in Asians, reporting that the *GSTM1* null genotype was associated with an increased CRC risk. Liao et al. [116] examined 23 studies including 5058 cases and 5999 controls to show that the *GSTT1* null genotype was associated with an increased CRC risk in Caucasians and Asians. Wan et al. [117] identified 30 studies of 7635 cases and 12,911 controls in all races, and demonstrated that the *GSTT1* null genotype was associated with an increased CRC risk in Caucasians. Teng et al. [118] examined 13 studies (including 2225 cases and 3990 controls) to assess the *GSTM1* null genotype with CRC risk and they found that the *GSTM1* null genotype was associated with an increased CRC risk in Chinese. Gao et al. [119] assessed the association of the *GSTM1* null genotype with CRC risk in all races (including 10,009 cases and 15,070 controls from 36 studies) and indicated that the *GSTM1* null genotype was associated with an increased risk of CRC, especially in Caucasians. Qin et al. [120] selected 46 studies including 15,373 cases and 21,238 controls to show that the *GSTT1* null genotype may contribute to an increased CRC risk in Asians and Caucasians. Wang et al. [121] (19 studies including 3130 cases and 6423 controls) found that the null genotypes of *GSTM1* and *GSTT1* and the dual null genotype of *GSTM1/GSTT1* were not associated with CRC risk in Chinese population. The examination of 44 studies of *GSTM1* (11,998 CRC cases and 17,552 controls) and 34 studies of *GSTT1* (8596 CRC cases and 13,589 controls) by Economopoulos and Sergentanis [122] indicated that the *GSTM1* and *GSTT1* null genotypes were associated with an increased CRC risk in Caucasians. Li et al. [123] analyzed 33 studies (including 8502 CRC Asian cases and 13,699 controls) and indicated that the *GSTM1* null genotype conferred susceptibility to CRC, especially in Chinese population. Xu et al. [124] examined 13 publications of 4832 cases and 7045 controls, demonstrating that the *GSTT1* null genotype was associated with an increased CRC risk in Asians. Zhong et al. [125] conducted an association of 12 studies involving 4517 cases and 6607 controls, and suggested that the *GSTT1* null genotype contributed to an increased CRC risk in Asians. Du et al. [126] examined 12 studies of *GSTM1* and 8 studies of *GSTT1*, and found no association on the *GSTM1* or *GSTT1* null genotype with CRC risk. Huang et al. [127] selected 55 studies including 17,498 cases and 26,441 controls to show that the *GSTM1* null genotype was a risk factor for CRC.

The current meta-analysis has several advantages over previous meta-analyses [115–125,126,127]. First, the sample size was much larger, with 86 case–control studies including 24,931 CRC cases and 36,537 controls evaluated for the *GSTM1* present/null polymorphism, 64 case–control studies including 19,725 CRC cases and 28,725 controls for the *GSTT1* present/null polymorphism, and 33 case-control studies including 8306 CRC cases and 14,369 controls for their combined effects in all races. Second, this is the first meta-analysis to explore their combined effects in overall population. Third, we used a meta-regression analysis method to explore the source of heterogeneity. Finally, the current meta-analysis included the most recent relevant publications to produce more accurate results.

Similar to previous meta-analyses, our study also has several limitations. First, only published articles were selected. Hence, publication bias may be found as shown in Figure 13. Moreover, positive results are known to be published more readily than negative ones. If negative results were included, an underestimation of the effect may be observed. Second, some case–control studies were based on hospital-based controls. These controls with non-cancerous disease may influence the pooled results in this study. Therefore, the use of population-based control studies may be more appropriate than hospital-based control studies. Third, only one study on Africans and Indians were included in the present study. Further new original studies were need on these issues in Africans and Indians.

In summary, the present study indicates that the *GSTM1* null genotype is associated with increased CRC risk in Asians and Caucasians, the *GSTT1* null genotype is associated with increased CRC risk in Asians, and the *GSTM1* null/*GSTT1* null genotype was associated with increased CRC risk in Asians. Further investigations involving large population-based studies should be conducted to explore the associations on the *GSTM1* null genotype, *GSTT1* null genotype and their combined effects with CRC risk.

## Data Availability

All relevant data are within the paper.

## Abbreviations

CBM: Chinese Biomedical Medical
CI: confidence interval
CNKI: China National Knowledge Infrastructure
CRC: colorectal cancer
GSTM1: glutathione S-transferase M1
GSTT1: glutathione S-transferase T1
MOOSE: Meta-analyses of Observational Studies in Epidemiology
OR: odds ratio
